# More green, less lonely? A longitudinal cohort study

**DOI:** 10.1093/ije/dyab089

**Published:** 2021-05-31

**Authors:** Thomas Astell-Burt, Terry Hartig, Simon Eckermann, Mark Nieuwenhuijsen, Anne McMunn, Howard Frumkin, Xiaoqi Feng

**Affiliations:** 1 Population Wellbeing and Environment Research Lab (PowerLab), School of Health and Society, Faculty of Arts, Social Sciences and Humanities, University of Wollongong, Wollongong, Australia; 2 Menzies Centre for Health Policy, School of Public Health, University of Sydney, Sydney, Australia; 3 National Institute of Environmental Health, China Center for Disease Control and Prevention (China CDC), Beijing, China; 4 School of Public Health, Peking Union Medical College and The Chinese Academy of Medical Sciences, Beijing, China; 5 Illawarra Health and Medical Research Institute (IHMRI), University of Wollongong, Wollongong, Australia; 6 Institute for Housing and Urban Research, Uppsala University, Uppsala, Sweden; 7 Department of Psychology, Uppsala University, Uppsala, Sweden; 8 School of Health and Society, Faculty of Arts, Social Sciences and Humanities, University of Wollongong, Wollongong, Australia; 9 ISGlobal, Barcelona, Spain; 10 Universitat Pompeu Fabra (UPF), Barcelona, Spain; 11 CIBER Epidemiología y Salud Pública (CIBERESP), Madrid, Spain; 12 Mary MacKillop Institute for Health Research, Melbourne, Australia; 13 UCL Department of Epidemiology & Public Health, UK; 14 Department of Environmental and Occupational Health Sciences, School of Public Health, University of Washington, Seattle, WA, USA; 15 School of Population Health, Faculty of Medicine and Health, The University of New South Wales, Sydney, Australia

**Keywords:** Loneliness, isolation, restoration, social contacts, parks, nature, cities, panel data, COVID-19

## Abstract

**Background:**

Urban greening may reduce loneliness by offering opportunities for solace, social reconnection and supporting processes such as stress relief. We (i) assessed associations between residential green space and cumulative incidence of, and relief from, loneliness over 4 years; and (ii) explored contingencies by age, sex, disability and cohabitation status.

**Methods:**

Multilevel logistic regressions of change in loneliness status in 8049 city-dwellers between 2013 (baseline) and 2017 (follow-up) in the Household, Income and Labour Dynamics in Australia study. Associations with objectively measured discrete green-space buffers (e.g. parks) (<400, <800 and <1600 m) were adjusted for age, sex, disability, cohabitation status, children and socio-economic variables. Results were translated into absolute risk reductions in loneliness per 10% increase in urban greening.

**Results:**

The absolute risk of loneliness rose from 15.9% to 16.9% over the 4 years; however, a 10% increase in urban greening within 1.6 km was associated with lower cumulative incident loneliness [odds ratio (OR) = 0.927, 95% confidence interval (CI) = 0.862 to 0.996; absolute risk reduction = 0.66%]. Stronger association was observed for people living alone (OR = 0.828, 95% CI = 0.725 to 0.944). In comparison to people with <10% green space, the ORs for cumulative incident loneliness were 0.833 (95% CI = 0.695 to 0.997), 0.790 (95% CI = 0.624 to 1.000) and 0.736 (95% CI = 0.549 to 0.986) for 10–20%, 20–30% and >30% green space, respectively. Compared with the <10% green-space reference group with 13.78% incident loneliness over 4 years and conservatively assuming no impact on incident loneliness, associations translated into absolute risk reductions of 1.70%, 2.26% and 2.72% within populations with 10–20%, 20–30% and >30% green space, respectively. These associations were stronger again for people living alone, with 10–20% (OR = 0.608, 95% CI = 0.448 to 0.826), 20–30% (OR = 0.649, 95% CI = 0.436 to 0.966) and >30% (OR = 0.480, 95% CI = 0.278 to 0.829) green space within 1600 m. No age, sex or disability-related contingencies, associations with green space within 400 or 800 m or relief from loneliness reported at baseline were observed.

**Conclusions:**

A lower cumulative incidence of loneliness was observed among people with more green space within 1600 m of home, especially for people living alone. Potential biopsychosocial mechanisms warrant investigation.

Key Messages

- Loneliness is a major risk factor for diabetes, heart disease, depression and premature death.
- There has been little empirical research on green space and loneliness, although anecdotal evidence from the COVID-19 pandemic suggests that people have flocked to parks to connect with others.
- Analyses of an Australian nationally representative cohort study suggest that achieving urban-greening targets of 30% total area could lower the odds of cumulative incident loneliness by up to 26% among adults in general.
- This 30% urban-greening target may lower the odds of cumulative incident loneliness by 52% among adults who live alone.
- Mechanisms linking green space with loneliness warrant investigation, as does the possibility that a reduction in the risk of loneliness is among the causal pathways between green space and non-communicable disease prevention.

## Introduction

Loneliness is an aversive state^1^ in which one feels deprived of connection, comradery and companionship. Many scientists (e.g. Jeste *et al*.^2^) describe a loneliness epidemic and this is (or ought to be) a major concern, with increasing evidence linking loneliness with elevated risks of depression,^3^ heart disease and stroke,^4^ inflammation,^5^ dementia^6^ and premature death,^7^ including suicide.^8^^,^^9^ Calls for a personalized approach to address loneliness have been made,^10^ but evidence so far suggests that many person-focused interventions have little or no effect.^11^^,^^12^

The US National Academies of Sciences, Engineering and Medicine recently called for research on policy options for reducing loneliness.^13^ This followed the launch of a loneliness strategy^14^ in the UK in 2018, which shifted the focus of potential intervention from the individual to the community context, including places outside of homes and workplaces (i.e. ‘Third Places’^15^) where people can meet. As the UK strategy noted, parks and other green spaces can be appealing and free-to-enter settings that enable nourishing past-times like birdwatching^16^ or dog walking,^17^ facilitating serendipitous encounters and transformative interactions that foster greater senses of belonging.^18^^,^^19^

Evidence that green-space availability reduces loneliness remains surprisingly limited.^20^^,^^21^ Yet, abundant ethnographic, experimental and epidemiological findings affirm that multiple mediating processes can work together with social (re)connection to ameliorate its aversive aspects.^22^^,^^23^ By affording aesthetic experiences, pleasant activities and psychological distance from demanding circumstances, green space can enhance mood,^24^ restore executive cognitive functioning^25^^,^^26^ and interrupt maladaptive rumination that sustains depression,^27^ concomitant with loneliness.^3^ Green space may also support life-affirming experiences in solitude for people experiencing the distress, distrust and lack of felt safety that characterizes loneliness.^1^ For example, ethnographic research suggests that a bench, tree or garden within a park can evoke comforting memories that provide solace^28^ and the joy of a warm embrace that some may feel they cannot get from other people.^29^ As a familiar place and as a representation of the natural world, green space may also evoke feelings of connection in turn associated with greater levels of happiness.^30^ Importantly, one does not necessarily require physical immersion within green space to avoid loneliness. Some psychological benefits have been shown to occur simply through a pleasant view of a park from a window.^31^^,^^32^ Furthermore, loneliness may also be reduced for a person who is housebound (e.g. due to disability or a long-term health condition) as a result of potentially greater collective levels of efficacy, cohesion and optimism spilling over from positive neighbourly interactions supported by green space.

With loneliness a rising concern, and with urban densification and the rise in single-occupant living often accompanied by the loss of urban green space and its resident wildlife,^33^ evidence of the potential benefits of green space for loneliness reduction could be timely and consequential. In this study, we contribute to the evidence base by: (i) estimating the extent to which green-space access in urban communities influences population incidence of, and relief from, loneliness over time; and (ii) assessing to what extent these associations are experienced disproportionately among people who are older, living alone, of a particular sex or living with a disability and/or limiting long-term health condition.

## Methods

### Data

A total of 8049 participants with survey responses in 2013 (‘baseline’ hereafter) and 2017 (‘follow-up’) were selected from the ‘Household, Income and Labour Dynamics in Australia’ (HILDA) survey—a nationally representative annual panel survey.^34^ These participants had complete outcome and covariate data and resided in major cities (Sydney, Melbourne, Brisbane, Perth, Adelaide, Australia Capital Territory/Canberra, plus other urban areas within the states of New South Wales, Victoria and Queensland). Those not lonely at baseline (*n* = 6766) comprised Sample 1 and those who were lonely (*n* = 1282) comprised Sample 2 (see Supplementary Table S1, available as Supplementary data at *IJE* online). The Australian Government Department of Social Services approved access to HILDA and the University of Wollongong HREC gave ethical approval for the study.

### Loneliness

Loneliness was measured with the same item in the self-complete survey at baseline and at follow-up: ‘I often feel very lonely’ (1 = Strongly disagree, 2 = Disagree, 3 = Somewhat disagree, 4 = Neither agree nor disagree, 5 = Somewhat agree, 6 = Agree, 7 = Strongly agree). This variable was dichotomized to distinguish those who agreed that they often felt very lonely (1 = responses 5–7) from those who felt ambivalent or not lonely to any degree (0 = responses 1–4). Cumulative incidence of new-onset loneliness over a 4-year period was observed among people not lonely at baseline (Sample 1). Relief from loneliness (also as cumulative incidence, although this term is not typically applied to relief) was observed among people lonely at baseline (Sample 2). Two sensitivity checks were conducted with loneliness alternatively defined as agreement or strong agreement with the statement (scores of 6 or 7) and as strong agreement only (a score of 7).

### Green space

Green space was measured as the percentage land cover within ‘crow-fly’ buffers of 400 m (≈1/4 mile), 800 m (≈1/2 mile) and 1600 m (≈1 mile) radii, roughly corresponding to common walking distances used in public health and urban planning.^35^ The population-weighted centroid of the Statistical Area 1 (SA1) of residence at baseline and follow-up for each participant was used to anchor each buffer. Each SA1 is the smallest geographical scale available in HILDA and is the smallest unit for the release of census data in Australia, with residential populations of between 200 and 800 people and an average of 400 people. Green-space data were sourced from the Australian Bureau of Statistics (ABS) classification of Mesh Blocks as ‘parkland’ in 2016,^36^ including parks, sports ovals, nature reserves and other protected or conserved areas, but not private gardens, farmland (given the focus on cities) or street trees (given the focus on discrete areas of land described as green spaces, rather than greenery). Each green-space variable was formatted with 1-unit increments equal to 10% green space. These variables were top-coded at >30% in descriptive analyses due to small numbers at higher levels.

### Confounding and modifiers of potential associations

Factors taken into account that could potentially influence loneliness and access to urban green space included age, sex, change or stability in cohabitation status between baseline and follow-up (married or cohabiting vs living alone) and baseline variables for highest educational qualification, annual household income (Australian dollars), time spent unemployed within the previous 12 months, the presence of children under 15 years old within the household, whether or not the participant self-reported living with a disability or long-term health condition that restricts every day activities and area-level socio-economic circumstances (measured using the ABS Index of Relative Socioeconomic Disadvantage^37^). Age, cohabitation status, sex and disability or long-term health condition were also considered potential modifiers of association between loneliness and green space.

### Statistical analysis

Patterns of loneliness incidence and relief across covariates were assessed using cross-tabulations. Separate multilevel logistic-regression models were developed in MLwiN^38^ to test the association between the incidence of, or relief from, loneliness and green-space exposures. Initial models adjusted for age, sex, children and cohabitation status, followed by further adjustment for socio-economic variables. All models took account of the clustering of participants within up to 194 Statistical Area 3s, which are geographic units built by the ABS from spatially contiguous SA1s with similar characteristics (residential populations of between 30 000 and 130 000) and designed to align closely with areas serviced by major transport and commercial hubs. There was an average of 34 study participants per SA3 (min = 1, max = 119). Modification was assessed by fitting two-way interaction terms for green space with (i) age, (ii) cohabitation status, (iii) sex and (iv) disability status, followed by stratified models for any interactions reaching statistical significance. Lastly, these results were translated to absolute risk reductions using a robust odds-ratio (OR) method (with steps provided in the ‘Results’ section).^39^

## Results

### Descriptive analyses

The cumulative incidence of loneliness was lowest among people in their mid-50s through mid-70s, with higher levels at younger and older ages (Table 1); higher among women and people living alone at both baseline and follow-up or at follow-up only; and lower for those with more green space within 1600 m. The incidence of loneliness was also higher for those with lower levels of education, lower incomes, living with disability or in more disadvantaged neighbourhoods (Supplementary Table S2, available as Supplementary data at *IJE* online). Levels of relief from loneliness tended to be highest among people initially living alone who then were married or cohabiting at follow-up and lowest among those initially married or cohabiting who were then living alone at follow-up. No clear pattern of relief from loneliness was observed across strata of green space (Table 1). Relief was more common with higher education, higher income, living without disability or in more affluent areas (Supplementary Table S2, available as Supplementary data at *IJE* online).

**Table 1:** Description of Samples 1 (incidence of loneliness) and 2 (relief from loneliness)

	Sample 1: Not lonely at baseline				Sample 2: Lonely at baseline		
	*N* not lonely at baseline	*n* lonely at follow-up	Cumulative incidence of loneliness % (95% confidence interval)		*N* lonely at baseline	*n* not lonely at follow-up	Cumulative relief from loneliness % (95% confidence interval)
Total	6766	817	12.08 (11.32 to 12.87)		1282	733	57.18 (54.45 to 59.86)
Age group (years)							
15–24	1100	151	13.73 (11.82 to 15.89)		210	126	60.00 (53.21 to 66.43)
25–34	1253	160	12.77 (11.03 to 14.74)		208	120	57.69 (50.85 to 64.25)
35–44	1204	155	12.87 (11.10 to 14.89)		219	120	54.79 (48.14 to 61.29)
45–54	1177	141	11.98 (10.24 to 13.96)		255	140	54.90 (48.73 to 60.92)
55–64	1018	90	8.84 (7.24 to 10.75)		208	110	52.88 (46.07 to 59.60)
65–74	690	67	9.71 (7.71 to 12.16)		123	77	62.60 (53.70 to 70.73)
75+	324	53	16.36 (12.71 to 20.80)		59	40	67.80 (54.81 to 78.51)
*p*(trend)			0.001				0.276
Sex							
Male	3195	352	11.02 (9.98 to 12.15)		536	313	58.40 (54.16 to 62.51)
Female	3571	465	13.02 (11.96 to 14.17)		746	420	56.30 (52.71 to 59.83)
*p*(trend)			0.012				0.455
Children (<15 years old) in the household							
No	4580	533	11.64 (10.74 to 12.60)		922	526	57.05 (53.82 to 60.22)
Yes	2186	284	12.99 (11.65 to 14.47)		360	207	57.50 (52.32 to 62.52)
*p*(trend)			0.110				0.884
Cohabitation status							
Married or cohabiting throughout	4341	416	9.58 (8.74 to 10.50)		569	366	64.32 (60.29 to 68.16)
Married or cohabiting, then living alone	282	66	23.40 (18.82 to 28.71)		83	38	45.78 (35.34 to 56.61)
Living alone, then married or cohabiting	429	34	7.93 (5.71 to 10.89)		85	61	71.76 (61.24 to 80.35)
Living alone throughout	1714	301	17.56 (15.83 to 19.44)		545	268	49.17 (44.98 to 53.38)
*p*(trend)			<0.001				<0.001
Green space within 1600 m							
0–10%	2460	339	13.78 (12.47 to 15.20)		463	264	57.02 (52.45 to 61.47)
10–20%	2504	287	11.46 (10.27 to 12.77)		496	285	57.46 (53.05 to 61.75)
20–30%	1099	122	11.10 (9.37 to 13.10)		198	112	56.57 (49.55 to 63.32)
>30%	703	69	9.82 (7.82 to 12.25)		125	72	57.60 (48.75 to 65.99)
*p*(trend)			0.007				0.996
Green space within 800 m							
0–10%	3023	399	13.20 (12.04 to 14.45)		541	310	57.30 (53.08 to 61.42)
10–20%	1962	224	11.42 (10.08 to 12.90)		413	229	55.45 (50.61 to 60.19)
20–30%	988	108	10.93 (9.13 to 13.04)		179	109	60.89 (53.53 to 67.79)
>30%	793	86	10.84 (8.86 to 13.21)		149	85	57.05 (48.95 to 64.78)
*p*(trend)			0.081				0.678
Green space within 400 m							
0–10%	3885	480	12.36 (11.36 to 13.43)		722	407	56.37 (52.72 to 59.95)
10–20%	1329	147	11.06 (9.48 to 12.86)		273	160	58.61 (52.65 to 64.32)
20–30%	898	114	12.69 (10.67 to 15.04)		145	85	58.62 (50.41 to 66.38)
>30%	654	76	11.62 (9.38 to 14.31)		142	81	57.04 (48.75 to 64.96)
*p*(trend)			0.567				0.909

Restricting the definition of loneliness to participants who either agreed or strongly agreed with the statement ‘I often feel very lonely’ (sensitivity 1) reduced the cumulative incidence of loneliness to 7.0% from 12.1% (prime definition, which included somewhat agreed). A lower cumulative incidence of loneliness was observable among participants with higher quantities of green space within 1600 m and to a lesser extent within 800 m, but not within 400 m (Supplementary Table S3, available as Supplementary data at *IJE* online). Tightening the definition exclusively to those who strongly agreed (sensitivity 2) reduced the cumulative incidence of loneliness to 2.6% and resulted in low response counts with no discernible pattern across strata of green space. The same restrictions applied to the assessment of relief from loneliness provided similar results (Supplementary Table S4, available as Supplementary data at *IJE* online). Accordingly, the prime definition of cumulative incidence of, and relief from, loneliness was focused on for the remainder of the investigation.

### Association between green space and loneliness outcomes

Association between green space within 1600 m and cumulative incident loneliness [OR = 0.910 for each 10% increment in green space, 95% confidence interval (CI) = 0.847 to 0.977] was observed after partial adjustment (Table 2, full results in Supplementary Table S5, available as Supplementary data at *IJE* online). This was slightly attenuated (OR = 0.927, 95% CI = 0.862 to 0.996) after full adjustment. Associations between green space within 800 and 400 m and cumulative incident loneliness did not reach statistical significance, although they were in the hypothesized direction. The odds of relief from loneliness tended to be higher with each 10% increment of green space within each buffer, but none of these associations reached statistical significance (Table 2, full results in Supplementary Table S6, available as Supplementary data at *IJE* online).

**Table 2:** Association between green space and cumulative incidence of, and relief from, loneliness

	Partially adjusted	Fully adjusted
	Odds ratio (95% confidence interval)	
Cumulative incidence of loneliness		
Percentage green space (10% units)		
within 1600 m	0.910 (0.847, 0.977)	0.927 (0.862, 0.996)
within 800 m	0.944 (0.885, 1.006)	0.957 (0.897, 1.021)
within 400 m	0.997 (0.944, 1.053)	1.007 (0.953, 1.063)
Cumulative relief from loneliness		
Percentage green space (10% units)		
within 1600 m	1.036 (0.937, 1.147)	1.027 (0.927, 1.139)
within 800 m	1.020 (0.929, 1.120)	1.015 (0.922, 1.116)
within 400 m	1.013 (0.935, 1.097)	1.008 (0.929, 1.094)

Partially adjusted = age group, sex, children, cohabitation status (time-varying).

Fully adjusted = age group, sex, children, cohabitation status (time-varying), highest educational qualifications, annual household income, % of last 12 months spent unemployed, disability or limiting long-term health condition, area-level socio-economic circumstances.

### Modification

A two-way interaction term (*p* = 0.030) indicated some degree of modification of the association between green space within 1600 m and cumulative incidence of loneliness by cohabitation status (Supplementary Table S7, available as Supplementary data at *IJE* online). No evidence of modification by cohabitation status was found for relief from loneliness, nor across age groups, by sex or disability status for either outcome. Fully adjusted stratified models indicated a lower odds of cumulative incidence of loneliness with more green space within 1600 m among participants living alone at baseline and follow-up (OR = 0.828, 95% CI = 0.725 to 0.944) (Table 3). The corresponding OR for people married or cohabiting at baseline and follow-up was weaker (OR = 0.938, 95% CI = 0.853 to 1.032, *p* = 0.189), although in the direction hypothesized. More green space was positively associated (*p* = 0.118) with cumulative incident loneliness among participants married or cohabiting at baseline but living alone at follow-up. The model for participants living alone at baseline and married or cohabiting at follow-up failed to converge; consequently, no results are reported.

**Table 3:** Association between green space within 1600 m and cumulative incidence of loneliness among people living alone at baseline and follow-up

	Stratified models
	Odds ratio (95% confidence interval)
Percentage green space (10% units)	
Living alone throughout	0.828 (0.725, 0.944)
Married or cohabiting throughout	0.938 (0.853, 1.032)
Married or cohabiting, then living alone	1.221 (0.950, 1.568)

Fully adjusted = age group, sex, children, cohabitation status (time-varying), highest educational qualifications, annual household income, % of last 12 months spent unemployed, disability or limiting long-term health condition, area-level socio-economic circumstances. Model did not converge and results are not shown for participants living alone at baseline and married or cohabiting at follow-up.

### Association between policy-relevant green-space targets and loneliness

**Figure 1:**
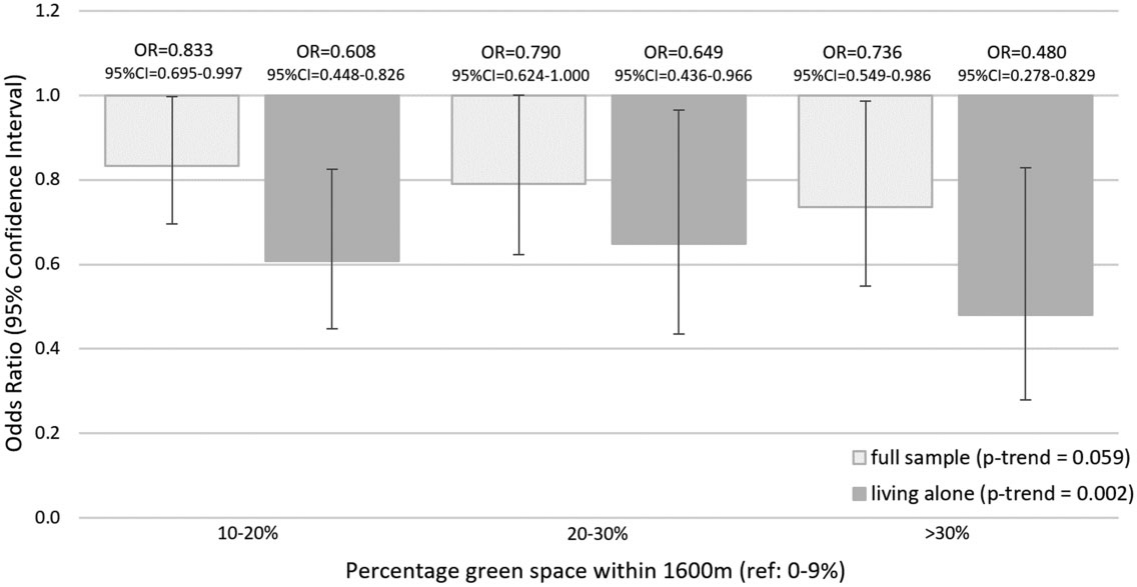
Association between green space within 1600 m and cumulative incidence of loneliness in all participants, and among those living alone at baseline and follow-up Both models were fully adjusted, including age group, sex, children, cohabitation status, percentage green space within 1600 m, highest educational qualifications, annual household income, % of last 12 months spent unemployed, disability or limiting long-term health condition, area-level socio-economic circumstances.

Contrasts in associations among all people and for people living alone at baseline and follow-up at policy-relevant levels of green-space exposure are shown in Figure 1. Compared with people with <10% green space within 1600 m, the ORs for cumulative incidence of loneliness were lower at 10–20% (OR = 0.833, 95% CI = 0.695 to 0.997), 20–30% (OR = 0.790, 95% CI = 0.624 to 1.000) and >30% (OR = 0.736, 95% CI = 0.549 to 0.986) levels of green space. Stronger odds ratios were observed among the subset of people living alone at baseline and follow-up at 10–20% (OR = 0.608, 95% CI = 0.448 to 0.826), 20–30% (OR = 0.649, 95% CI = 0.436 to 0.966) and >30% (OR = 0.480, 95% CI = 0.278 to 0.829) levels of green space within 1600 m.

### Absolute risk reductions

The 0.927 OR for cumulative-loneliness incidence associated with a 10% difference in green space translates into a 0.66% absolute risk reduction across community populations over the 4-year study period in the prevalence of loneliness. This estimate was calculated using robust OR methods for evidence translation of incident ORs^39^ to observed risk over 4 years as per the steps below. The association of greater relief (OR = 1.027, 95% CI = 0.927 to 1.139, *p* = 0.608) from loneliness with 10% more green space in the 15.9% prevalent population at baseline in Sample 2 was conservatively assumed null in estimation.

1. The baseline cumulative incidence of loneliness in Sample 1 (*n* = 817 from 6766 people not lonely at baseline, see Table 2) was converted from a probability (0.121 = 817/6766) to an odds [0.137 = 0.121/(1 – 0.121)].
2. This baseline study odds of cumulative incident loneliness in Sample 1 was then multiplied by the OR to estimate the odds from a 10% increase in green space within 1600 m (0.127 = 0.137 × 0.927).
3. This odds was converted into a probability [0.113 = 0.127/(1 + 0.127)] of cumulative incident loneliness.
4. The absolute risk reduction of cumulative loneliness was estimated for Sample 1 subtracting that with 10% additional greening (0.113) from the baseline cumulative incidence of loneliness probability (0.121) to estimate the absolute reduction from baseline in cumulative incident loneliness (0.00793 = 0.121 – 0.113).
5. Finally, to estimate the absolute risk reduction at the community level, the risk reduction in the cumulative incident population was combined with that in the prevalent population (conservatively assumed 0). This involved multiplying the absolute risk reduction of 0.00793 in step 4 by the proportion of Sample 1 that were not lonely at baseline [0.0067 = 0.00793 ×  (6766/8049)]. The 0.0066 is then reported as a percentage (0.66%).

These steps were also used to calculate the absolute risk reductions in the cumulative incidence of loneliness over 4 years with respect to ORs contrasting policy-relevant targets for green space within 1600 m (Figure 1). Compared with a community with <10% green space (with a 13.78% baseline risk of incident loneliness and a prevalence of 15.9% loneliness at baseline), the absolute risk reduction in the cumulative incidence of loneliness over 4 years was 1.70% at 10–20% green space within 1600 m. The absolute risk reductions for 20–30% and >30% green space within 1600 m compared with the same <10% reference group were 2.26% and 2.72%, respectively.

## Discussion

This longitudinal cohort study found an association between 10% increments in urban green space within 1600 m and lower cumulative incidence of loneliness over 4 years. This association was especially strong among people living alone at baseline and follow-up. Associations between green space within shorter distances and cumulative incident loneliness were comparatively weaker. No convincing evidence of association was observed between green space and relief from loneliness. Although biopsychosocial mechanisms still require elucidation, these results further encourage a view of urban greening as a population-level intervention for the potential prevention of loneliness. Moreover, translation of our findings in absolute risk reductions indicated a targeted strategy focusing investments in urban greening within communities containing <10% green space could result in greater reductions in the risk of loneliness.

These results are tentative given the observational nature of the data and the likelihood of residual confounding, but they do build meaningfully upon previous studies of cross-sectional design^20^^,^^21^ by ensuring that the exposure preceded the outcome. Identical questions on loneliness at baseline and follow-up enabled separate analyses of the cumulative incidence of, and relief from, loneliness that were not possible in previous cross-sectional studies. These were answered in a self-complete questionnaire filled in separately from interviews.

It is important to acknowledge that loneliness is neither an illness nor a diagnosis and that it is multifaceted in ways obscured by the single-item measure available in this study. For example, insight was not possible into specific types of loneliness that may be more or less amenable to environmental intervention (e.g. social loneliness, romantic loneliness, familial estrangement). Future work with multidimensional measures of loneliness, such as the multi-item UCLA scale^40^ or the De Jong Gierveld Loneliness Scale,^41^ will help to advance this new area of research. So too might studies designed to enable insights into ‘existential loneliness’—a felt situation concordant with feelings of despair, hopelessness and loss of meaning in life^42^^,^^43^ that may underpin pathological pathways resulting in noted increases in deaths from suicide, drug overdose and alcoholism.^44^ The limitations of the single-item general measure of loneliness used in the current study indicate that the estimates of association that we have presented are conservative and may account for the asymmetry of associations between green space and cumulative incidence of, and relief from, loneliness. The possibility that green space may ameliorate or aggravate different types of loneliness to different degrees via contrasting biopsychosocial mechanisms warrants examination.

Findings from previous studies suggest social connection and solace as two potential mechanisms. If, as Cacioppo *et al.*^1^ suggest, loneliness acts like hunger, thirst or pain to prompt behavioural change, then nearby green spaces could be settings that enable the restoration of feelings of social connection. Urban parks in particular were highlighted in interviews by Neal and colleagues^18^ as spaces where neighbours and families across multiple generations not only enjoyed meeting each other, but also felt reassured that there would be someone in the park whom they knew they could go to meet. Thus, green spaces within cities may support the formation and maintenance of social capital that guards against the development of loneliness and provides other positive network multiplier effects.^45^ Regular social programming can offer ways to reinforce a sense of community with green spaces as social hubs. Parks and other types of green space within cities often serve as settings for public gatherings and ritual events that sustain a sense of community, through which those spaces can become invested with individual as well as socially shared meanings (e.g.^46^). That said, feelings of alienation and of being ‘out of place’ are possible consequences of living within a neighbourhood with strong levels of bonding social capital when one is not part of the ‘in-group’, rendered painfully visible by how patrons interact with local green spaces in ways that leave some people feeling excluded (e.g. due to their ethnicity^47^).

Also plausible, though perhaps more difficult to measure, is the extent to which people ‘lean on green’ as an alternative hypothesized pathway from green space to reduced risk of loneliness. Research by Birch and colleagues in particular^29^ has revealed how some people prefer to seek out contact with nature absent other humans. Their interviewees cited green spaces as offering non-judgemental and dependable sources of support, especially when friends were not around (or were found wanting). These relationships with the ‘more-than-human-world’ may reflect the influence of attachment to places previously lived in,^28^ comforting memories developed earlier in the lifecourse^48^ and an otherwise positive attitude towards the natural world.^30^ Solace provided by the natural setting may work through intermediate individual outcomes previously mentioned (enhanced mood,^24^ restored executive cognitive functioning^25^^,^^26^ and interrupted maladaptive rumination^27^) to enable and enhance positive interactions with other nature-seekers sharing similar circumstances.^49^ Such crossover of benefits between individuals in green spaces may have special utility for reducing loneliness among people who, with higher levels of ‘nature-relatedness’ or personality characteristics such as introversion, seek momentary distance from problematic social interactions elsewhere. Research that examines the potential interplay between environmental attitudes or personality, access to green space and loneliness over time could help to qualify the extent to which this mechanism is likely.

Evidence of association was found between loneliness and green space within 1600 m, but not 800 or 400 m. Intuitively, green space nearby may be expected to be more strongly associated with loneliness than that located further afield, perhaps especially for the elderly and people with mobility difficulties. Several contextual features may have engendered these seemingly counter-intuitive results. First, exposure was based upon discrete green space within cities but outside of the household lot. People may have compensated for lower levels of green-space exposure within 400 or 800 m in our study through the use of backyards. Second, the green-space variables did not include shade and the aesthetic pleasure derived from street trees, which have been previously reported to support social capital formation^50^ and better mental health.^51^ Neighbourhoods with an abundance of street-tree canopy but no park or nature reserve may provide similar levels of support for reducing the risk of loneliness. Third, large quantities of discrete green space within close proximity to home may offer opportunities for connection and solace for some people, but may also increase physical isolation and a lack of felt safety if sparsely populated and poorly served by public infrastructure. Fourth, the cumulative opportunities for green space to activate pathways that support reductions in the risk of loneliness may be subject to exposure misclassification if relatively short distances are relied upon to the exclusion of green spaces located a little farther afield, which may be the case for larger parks that are more attractive for walking and other forms of recreation.^52^ Further to this point, some degree of substitution may also occur if one lacks access to green space at home but has ample availability in other contexts, such as near the workplace, school or preferred shopping location. Future work might consider examining multiple settings and implications for related issues (e.g. duration of exposure).

These issues around exposure point to the related and similarly important matter of heterogeneity through space and time. Recent work has indicated that only momentary views of greenery (e.g. a pleasant landscape through a window) can be sufficient for sustaining some psychological benefits of green space.^53^ Moreover, serendipitous encounters and transformative interactions can occur quickly, opening up new social interactions that play out across settings other than the green space where the initial contact occurred. In this study, we have made no assumptions around the duration of exposure to green space. However, research on effect modification is a potentially consequential avenue for future investigation, given that people may face competing demands for attention wherever they go. The proliferation of smartphones and the use of ‘social media’ have augmented the ways in which people spend time in solitude and interacting with others. Smartphones and social media have been associated with both increased *and* decreased risks of loneliness^54^ and diminished restorative benefits from nature contact.^55^ The impacts of social media on how people use their time and interact with—and within—green spaces and the amelioration of loneliness could be important avenues for exploration.

Other contingencies might include changes in green-space availability and quality resulting from relocation and via processes of urban regeneration and densification. Our study was restricted to a lagged measure of green-space quantity, with a change in availability through residential relocation omitted to minimize potential reverse causality. Changes in green-space availability and quality *in situ* were not available for analysis. A loss of green space and/or decline in its quality may symbolize felt secular changes in the wider context, a sense of loss of community, a decline in social capital and a potential trigger for loneliness.^56^ Changes in green space could prove important enablers for social connectedness, opening opportunities to meet and engage with new people from different sociocultural backgrounds. Therefore, an expanded longitudinal analysis may be valuable to test the potential for moderated mediation across contexts of community change.^57^ Such work is needed to inform investment in randomized–controlled trials and evaluations of natural experiments as additional components of evidence triangulation on policy options for loneliness prevention.^58^

In conclusion, loneliness is an aversive state in which people may have heightened sensitivity to, and may disproportionately reap rewards from, positive interventions that increase the inclusivity and socially as well as individually restorative nature of the neighbourhoods in which they live. When one considers the lack of alternative effective person-level interventions,^11^^,^^12^ our study suggests that urban greening could be an important population-level strategy for reducing the risk of loneliness. Investment in urban green space is already part of one national loneliness strategy^14^ and would likely complement—perhaps synergize with—other interventions as they are discovered and validated. It generally requires no entrance fee, no purchase of some good and/or service, likely appreciates in cultural, aesthetic and physical (e.g. shade) value to local communities through time and has well-documented co-benefits for mental and physical health^23^ and the environment (e.g. mitigation of air pollution^59^ and heat^60^). Addressing disparities in the availability of green space could, therefore, bolster a wide range of societal imperatives and, quite possibly, increase resilience to loneliness ahead of major shocks to societies. This possibility appears all the more plausible in light of evidence of urban residents’ turning to local green spaces in greater numbers during the present COVID-19 pandemic.^61–64^ This is a hitherto under-explored potential loneliness-prevention policy option that warrants investment informed by new high-quality observational studies, randomized trials and cost-effectiveness studies contrasting green-space restoration and maintenance costs with healthcare and wider societal costs saved, to reveal the full benefits of creating, conserving and equalizing access to green spaces across urban communities.

## Supplementary data

Supplementary data are available at *IJE* online.

## Ethics approval

The University of Wollongong HREC gave ethical approval for the study (2019/216).

## Funding

This study was supported by a National Health and Medical Research Council Boosting Dementia Research Leader Fellowship 1140317 (Astell-Burt) and National Health and Medical Research Council Career Development Fellowship 1148792 (Feng). Astell-Burt and Feng were also jointly supported by grant 1101065 from the National Health and Medical Research Council project and grant GC15005 from the Green Cities Fund - Hort Innovation Limited, with coinvestment from the University of Wollongong Faculty of Social Sciences, the University of Wollongong Global Challenges initiative, and the Australian Government. McMunn was supported by the UK Economic and Social Research Council International Centre for Lifecourse Studies in Society and Health (ICLS) [grant number ES/J019119/1]. The funding sources had no role in the design and conduct of the study; collection, management, analysis, and interpretation of the data; preparation, review, or approval of the manuscript; and the decision to submit the manuscript for publication.

## Data availability

The data underlying this article were provided by the Melbourne Institute, University of Melbourne under licence.

## Supplementary Material

dyab089_Supplementary_Data
