# Supplementary material for: More green, less lonely? A longitudinal cohort study
Source: Int J Epidemiol. 2021 May 31;51(1):99–110. doi: 10.1093/ije/dyab089 (PMC8194954; doi:10.1093/ije/dyab089)
Supplement: dyab089_Supplementary_Data [file dyab089_supplementary_data.docx]

**Table S1: Sample derivation**

|  |  |  |  |  |  |  |
| --- | --- | --- | --- | --- | --- | --- |
| **Waves** | **N** | **%** |  |  |  |  |
| Wave 13 (Baseline) | 17,482 | 49.90 |  |  |  |  |
| Wave 17 (Follow-up) * | 17,549 | 50.10 |  |  |  |  |
|  |  |  |  |  |  |  |
| **Survey responses: in sample** | **N** | **%** |  | **N** | **%** | **Survey responses: omitted** |
| Present in Baseline and Follow-up | 14,624 | 71.66% |  | 2,858 | 14.00 | Present in Baseline but not Follow-up |
|  |  |  |  | 2,925 | 14.33 | Present in Follow-up but not Baseline |
|  |  |  |  |  |  |  |
| **Geographic Remoteness: in sample** | **N** | **%** |  | **N** | **%** | **Geographic Remoteness: omitted** |
| Participants in 'Major Cities' | 9,674 | 66.15 |  | 3,191 | 21.82 | Participants in 'Inner Regional' areas |
|  |  |  |  | 1,543 | 10.55 | Participants in 'Outer Regional' areas |
|  |  |  |  | 216 | 1.48 | Participants in 'Remote' areas |
|  |  |  |  |  |  |  |
| **Distribution of participants within 'Major Cities'** | **N** | **%** |  |  |  |  |
| Sydney (NSW) | 2,381 | 24.61 |  |  |  |  |
| Melbourne (Victoria) | 2,524 | 26.09 |  |  |  |  |
| Brisbane (Queensland) | 1,317 | 13.61 |  |  |  |  |
| Adelaide (South Australia) | 910 | 9.41 |  |  |  |  |
| Perth (Western Australia) | 1,025 | 10.60 |  |  |  |  |
| Canberra (Australia Capital Territory) | 308 | 3.18 |  |  |  |  |
| Other major cities in NSW | 570 | 5.89 |  |  |  |  |
| Other major cities in Victoria | 105 | 1.09 |  |  |  |  |
| Other major cities in Queensland | 534 | 5.52 |  |  |  |  |
|  |  |  |  |  |  |  |
| **Baseline: I often feel very lonely: in sample** | **N** | **%** |  | **N** | **%** | **Baseline: I often feel very lonely: omitted** |
| [1] Strongly disagree | 3,324 | 34.36 |  | 941 | 9.73 | [-8] No Self-Complete Questionnaire (SCQ) |
| 2 | 2,157 | 22.30 |  | 10 | 0.10 | [-5] Multiple response to SCQ |
| 3 | 888 | 9.18 |  | 45 | 0.47 | [-4] Refused/Not stated |
| 4 | 912 | 9.43 |  |  |  |  |
| 5 | 603 | 6.23 |  |  |  |  |
| 6 | 479 | 4.95 |  |  |  |  |
| [7] Strongly agree | 315 | 3.26 |  |  |  |  |
|  |  |  |  |  |  |  |
| **Follow-up: I often feel very lonely: in sample** | **N** | **%** |  | **N** | **%** | **Follow-up: I often feel very lonely: omitted** |
| [1] Strongly disagree | 3,252 | 33.62 |  | 652 | 6.74 | [-8] No SCQ |
| 2 | 2,137 | 22.09 |  | 16 | 0.17 | [-5] Multiple response to SCQ |
| 3 | 895 | 9.25 |  | 152 | 1.57 | [-4] Refused/Not stated |
| 4 | 1,034 | 10.69 |  |  |  |  |
| 5 | 683 | 7.06 |  |  |  |  |
| 6 | 537 | 5.55 |  |  |  |  |
| [7] Strongly agree | 316 | 3.27 |  |  |  |  |
|  |  |  |  |  |  |  |
| **Loneliness transitions: sample 1** | **N** | **%** |  | **N** | **%** | **Loneliness transitions: sample 2** |
| Baseline not lonely -> Follow-up not lonely | 6,061 | 87.90 |  | 759 | 57.28 | Baseline lonely -> Follow-up not lonely |
| Baseline not lonely -> Follow-up lonely | 834 | 12.10 |  | 566 | 42.72 | Baseline lonely -> Follow-up lonely |
| Total | 6,895 |  |  | 1,325 |  | Total |
|  |  |  |  |  |  |  |
| **Covariates: omitted due to missing data** | **N** | **%** |  | **N** | **%** | **Covariates: omitted due to missing data** |
| % of last 12 months unemployed (baseline) | 1 | 0.01 |  | 40 | 3.02 | Annual household income (baseline) |
| Annual household income (baseline) | 125 | 1.81 |  | 1 | 0.01 | Highest educational qualification |
| Highest educational qualification | 1 | 0.01 |  | 1 | 0.01 | Area-level socioeconomic circumstances (baseline) |
| Cohabitation status (follow-up) | 1 | 0.01 |  | 1 | 0.01 | Disability or long-term health condition (baseline) |
| Area-level socioeconomic circumstances (baseline) | 1 | 0.01 |  | 42 | 3.17 | Total omitted due to missing covariate data |
| Total omitted due to missing covariate data | 128 | 1.86 |  |  |  |  |
| * Note: no missing data for age, sex, green space | |  |  |  |  |  |
|  |  |  |  |  |  |  |
| **Final sample 1** | **N** | **%** |  | **N** | **%** | **Final sample 2** |
| Baseline not lonely -> Follow-up not lonely | 5,949 | 87.92 |  | 549 | 42.82 | Baseline lonely -> Follow-up lonely |
| Baseline not lonely -> Follow-up lonely | 817 | 12.08 |  | 733 | 57.18 | Baseline lonely -> Follow-up not lonely |
| Total | 6,766 |  |  | 1,282 |  | Total |
| * Note: The sample size was greater in wave 17 (compared to wave 13) due to returning participants in the HILDA panel survey who did not participate in wave 13 | | | | | | |

**Table S2: Description of samples 1 (incidence of loneliness) and 2 (relief from loneliness)**

|  |  |  |  |  |  |  |  |
| --- | --- | --- | --- | --- | --- | --- | --- |
|  | Sample 1: Not lonely at baseline | | |  | Sample 2: Lonely at baseline | | |
|  | N not lonely at baseline | n lonely at follow-up | Cumulative incidence of loneliness % (95% Confidence Interval) |  | N lonely at baseline | n not lonely at follow-up | Cumulative relief from loneliness % (95% Confidence Interval) |
| Total | 6,766 | 817 | 12.08 (11.32 to 12.87) | | 1,282 | 733 | 57.18 (54.45 to 59.86) |
| Age group | | | | | | | |
| 15-24 | 1,100 | 151 | 13.73 (11.82 to 15.89) | | 210 | 126 | 60.00 (53.21 to 66.43) |
| 25-34 | 1,253 | 160 | 12.77 (11.03 to 14.74) | | 208 | 120 | 57.69 (50.85 to 64.25) |
| 35-44 | 1,204 | 155 | 12.87 (11.10 to 14.89) | | 219 | 120 | 54.79 (48.14 to 61.29) |
| 45-54 | 1,177 | 141 | 11.98 (10.24 to 13.96) | | 255 | 140 | 54.90 (48.73 to 60.92) |
| 55-64 | 1,018 | 90 | 8.84 (7.24 to 10.75) |  | 208 | 110 | 52.88 (46.07 to 59.60) |
| 65-74 | 690 | 67 | 9.71 (7.71 to 12.16) |  | 123 | 77 | 62.60 (53.70 to 70.73) |
| 75+ | 324 | 53 | 16.36 (12.71 to 20.80) | | 59 | 40 | 67.80 (54.81 to 78.51) |
| p(trend) |  |  | 0.001 |  |  |  | 0.276 |
| Sex | | | | | | | |
| Male | 3,195 | 352 | 11.02 (9.98 to 12.15) |  | 536 | 313 | 58.40 (54.16 to 62.51) |
| Female | 3,571 | 465 | 13.02 (11.96 to 14.17) | | 746 | 420 | 56.30 (52.71 to 59.83) |
| p(trend) |  |  | 0.012 |  |  |  | 0.455 |
| Children (<15y) in the household | | | | | | | |
| No | 4,580 | 533 | 11.64 (10.74 to 12.60) | | 922 | 526 | 57.05 (53.82 to 60.22) |
| Yes | 2,186 | 284 | 12.99 (11.65 to 14.47) | | 360 | 207 | 57.50 (52.32 to 62.52) |
| p(trend) |  |  | 0.110 |  |  |  | 0.884 |
| Cohabitation status | | | | | | | |
| Married or cohabiting throughout | 4,341 | 416 | 9.58 (8.74 to 10.50) |  | 569 | 366 | 64.32 (60.29 to 68.16) |
| Married or cohabiting, then living alone | 282 | 66 | 23.40 (18.82 to 28.71) | | 83 | 38 | 45.78 (35.34 to 56.61) |
| Living alone, then married or cohabiting | 429 | 34 | 7.93 (5.71 to 10.89) |  | 85 | 61 | 71.76 (61.24 to 80.35) |
| Living alone throughout | 1,714 | 301 | 17.56 (15.83 to 19.44) | | 545 | 268 | 49.17 (44.98 to 53.38) |
| p(trend) |  |  | <0.001 |  |  |  | <0.001 |
| Green space within 1600m | | | | | | | |
| 0-10% | 2,460 | 339 | 13.78 (12.47 to 15.20) | | 463 | 264 | 57.02 (52.45 to 61.47) |
| 10-20% | 2,504 | 287 | 11.46 (10.27 to 12.77) | | 496 | 285 | 57.46 (53.05 to 61.75) |
| 20-30% | 1,099 | 122 | 11.10 (9.37 to 13.10) |  | 198 | 112 | 56.57 (49.55 to 63.32) |
| >30% | 703 | 69 | 9.82 (7.82 to 12.25) |  | 125 | 72 | 57.60 (48.75 to 65.99) |
| p(trend) |  |  | 0.007 |  |  |  | 0.996 |
| Green space within 800m | | | | | | | |
| 0-10% | 3,023 | 399 | 13.20 (12.04 to 14.45) | | 541 | 310 | 57.30 (53.08 to 61.42) |
| 10-20% | 1,962 | 224 | 11.42 (10.08 to 12.90) | | 413 | 229 | 55.45 (50.61 to 60.19) |
| 20-30% | 988 | 108 | 10.93 (9.13 to 13.04) |  | 179 | 109 | 60.89 (53.53 to 67.79) |
| >30% | 793 | 86 | 10.84 (8.86 to 13.21) |  | 149 | 85 | 57.05 (48.95 to 64.78) |
| p(trend) |  |  | 0.081 |  |  |  | 0.678 |
| Green space within 400m | | | | | | | |
| 0-10% | 3,885 | 480 | 12.36 (11.36 to 13.43) | | 722 | 407 | 56.37 (52.72 to 59.95) |
| 10-20% | 1,329 | 147 | 11.06 (9.48 to 12.86) |  | 273 | 160 | 58.61 (52.65 to 64.32) |
| 20-30% | 898 | 114 | 12.69 (10.67 to 15.04) | | 145 | 85 | 58.62 (50.41 to 66.38) |
| >30% | 654 | 76 | 11.62 (9.38 to 14.31) |  | 142 | 81 | 57.04 (48.75 to 64.96) |
| p(trend) |  |  | 0.567 |  |  |  | 0.909 |
| Highest educational qualifications | | | | | | | |
| < Year 12 | 1,504 | 216 | 14.36 (12.68 to 16.23) | | 355 | 192 | 54.08 (48.86 to 59.22) |
| Year 12 to Adv. Dip. | 3,082 | 395 | 12.82 (11.68 to 14.04) | | 611 | 351 | 57.45 (53.48 to 61.32) |
| University | 2,180 | 206 | 9.45 (8.29 to 10.75) |  | 316 | 190 | 60.13 (54.61 to 65.40) |
| p(trend) |  |  | <0.001 |  |  |  | 0.283 |
| Annual household income | | | | | | | |
| 0-$30k | 624 | 110 | 17.63 (14.83 to 20.82) | | 203 | 110 | 54.19 (47.27 to 60.94) |
| $30k-$40k | 439 | 79 | 18.00 (14.67 to 21.88) | | 100 | 53 | 53.00 (43.17 to 62.60) |
| $40k-$50k | 420 | 64 | 15.24 (12.11 to 19.01) | | 106 | 55 | 51.89 (42.37 to 61.27) |
| $50k-$60k | 468 | 60 | 12.82 (10.08 to 16.17) | | 104 | 57 | 54.81 (45.13 to 64.14) |
| $60k-$80k | 695 | 82 | 11.80 (9.60 to 14.42) |  | 162 | 84 | 51.85 (44.14 to 59.47) |
| $80k-$100k | 750 | 86 | 11.47 (9.37 to 13.95) |  | 130 | 65 | 50.00 (41.45 to 58.55) |
| $100k-$125k | 851 | 90 | 10.58 (8.68 to 12.83) |  | 150 | 96 | 64.00 (55.98 to 71.30) |
| 125k+ | 2,519 | 246 | 9.77 (8.67 to 10.99) |  | 327 | 213 | 65.14 (59.80 to 70.12) |
| p(trend) |  |  | <0.001 |  |  |  | 0.009 |
| % of last 12 months spent unemployed | | | | | | | |
| 0% | 6,169 | 741 | 12.01 (11.22 to 12.85) | | 1,087 | 625 | 57.50 (54.53 to 60.41) |
| 1-24% | 261 | 30 | 11.49 (8.15 to 15.98) |  | 61 | 29 | 47.54 (35.31 to 60.08) |
| 25-49% | 127 | 17 | 13.39 (8.47 to 20.52) |  | 50 | 32 | 64.00 (49.79 to 76.12) |
| 50-74% | 93 | 9 | 9.68 (5.09 to 17.62) |  | 23 | 13 | 56.52 (35.86 to 75.14) |
| 75-100% | 116 | 20 | 17.24 (11.38 to 25.26) | | 61 | 34 | 55.74 (43.06 to 67.71) |
| p(trend) |  |  | 0.444 |  |  |  | 0.499 |
| Disability or long-term health condition |  |  |  |  |  |  |  |
| no | 5,129 | 560 | 10.92 (10.09 to 11.80) | | 786 | 484 | 61.58 (58.12 to 64.92) |
| yes | 1,637 | 257 | 15.70 (14.02 to 17.54) | | 496 | 249 | 50.20 (45.80 to 54.60) |
| p(trend) |  |  | <0.001 |  |  |  | <0.001 |
| Area-level socioeconomic circumstances (deciles) | | | | | | | |
| 1 | 1,086 | 114 | 10.50 (8.81 to 12.47) |  | 155 | 101 | 65.16 (57.31 to 72.27) |
| 2 | 901 | 88 | 9.77 (7.99 to 11.89) |  | 147 | 83 | 56.46 (48.31 to 64.28) |
| 3 | 923 | 85 | 9.21 (7.50 to 11.25) |  | 167 | 101 | 60.48 (52.85 to 67.63) |
| 4 | 791 | 105 | 13.27 (11.08 to 15.82) | | 142 | 87 | 61.27 (52.98 to 68.95) |
| 5 | 721 | 86 | 11.93 (9.76 to 14.51) |  | 120 | 66 | 55.00 (45.99 to 63.70) |
| 6 | 618 | 84 | 13.59 (11.11 to 16.53) | | 116 | 65 | 56.03 (46.85 to 64.82) |
| 7 | 498 | 71 | 14.26 (11.45 to 17.62) | | 108 | 61 | 56.48 (46.96 to 65.55) |
| 8 | 488 | 53 | 10.86 (8.39 to 13.95) |  | 118 | 63 | 53.39 (44.33 to 62.23) |
| 9 | 418 | 71 | 16.99 (13.68 to 20.90) | | 114 | 59 | 51.75 (42.58 to 60.81) |
| 10 | 322 | 60 | 18.63 (14.74 to 23.27) | | 95 | 47 | 49.47 (39.51 to 59.48) |
| p(trend) |  |  | <0.001 |  |  |  | 0.315 |
|  |  |  |  |  |  |  |  |

**Table S3: Pattern of cumulative incidence of loneliness of contrasting definitions across green space strata**

|  |  |  |  |  |  |  |  |  |  |  |  | |
| --- | --- | --- | --- | --- | --- | --- | --- | --- | --- | --- | --- | --- |
| Green space | Cumulative incidence of loneliness (prime definition) | | |  | Cumulative incidence of loneliness (sensitivity 1) | | |  | Cumulative incidence of loneliness (sensitivity 2) | | | |
|  | N total | N cumulative incidence | % (95% Confidence Interval) |  | N total | N cumulative incidence | % (95% Confidence Interval) |  | N total | N cumulative incidence | % (95% Confidence Interval) | |
| Total | 6,766 | 817 | 12.1 (11.3 to 12.9) |  | 7,319 | 510 | 7.0 (6.4 to 7.6) |  | 7,764 | 205 | 2.6 (2.3 to 3.0) | |
| Percent green space within 1600m | | | | | | | | | | | |  |
| 0-10% | 2,460 | 339 | 13.8 (12.5 to 15.2) |  | 2,677 | 212 | 7.9 (7.0 to 9.0) |  | 2,823 | 80 | 2.8 (2.3 to 3.5) | |
| 10-20% | 2,504 | 287 | 11.5 (10.3 to 12.8) |  | 2,712 | 170 | 6.3 (5.4 to 7.2) |  | 2,891 | 75 | 2.6 (2.1 to 3.2) | |
| 20-30% | 1,099 | 122 | 11.1 (9.4 to 13.1) |  | 1,177 | 85 | 7.2 (5.9 to 8.8) |  | 1,255 | 32 | 2.5 (1.8 to 3.6) | |
| >30% | 703 | 69 | 9.8 (7.8 to 12.2) |  | 753 | 43 | 5.7 (4.3 to 7.6) |  | 795 | 18 | 2.3 (1.4 to 3.6) | |
| p(trend) |  |  | 0.007 |  |  |  | 0.052 |  |  |  | 0.822 | |
|  |  |  |  |  |  |  |  |  |  |  |  | |
| Percent green space within 800m | | | | | | | | | | | |  |
| 0-10% | 3,023 | 399 | 13.2 (12.0 to 14.5) |  | 3,259 | 229 | 7.0 (6.2 to 8.0) |  | 3,444 | 86 | 2.5 (2.0 to 3.1) | |
| 10-20% | 1,962 | 224 | 11.4 (10.1 to 12.9) |  | 2,129 | 156 | 7.3 (6.3 to 8.5) |  | 2,279 | 67 | 2.9 (2.3 to 3.7) | |
| 20-30% | 988 | 108 | 10.9 (9.1 to 13.0) |  | 1,072 | 70 | 6.5 (5.2 to 8.2) |  | 1,128 | 25 | 2.2 (1.5 to 3.3) | |
| >30% | 793 | 86 | 10.8 (8.9 to 13.2) |  | 859 | 55 | 6.4 (4.9 to 8.2) |  | 913 | 27 | 3.0 (2.0 to 4.3) | |
| p(trend) |  |  | 0.081 |  |  |  | 0.757 |  |  |  | 0.529 | |
|  |  |  |  |  |  |  |  |  |  |  |  | |
| Percent green space within 400m | | | | | | | | | | | |  |
| 0-10% | 3,885 | 480 | 12.4 (11.4 to 13.4) |  | 4,181 | 296 | 7.1 (6.3 to 7.9) |  | 4,440 | 117 | 2.6 (2.2 to 3.1) | |
| 10-20% | 1,329 | 147 | 11.1 (9.5 to 12.9) |  | 1,455 | 92 | 6.3 (5.2 to 7.7) |  | 1,545 | 32 | 2.1 (1.5 to 2.9) | |
| 20-30% | 898 | 114 | 12.7 (10.7 to 15.0) |  | 969 | 69 | 7.1 (5.7 to 8.9) |  | 1,006 | 29 | 2.9 (2.0 to 4.1) | |
| >30% | 654 | 76 | 11.6 (9.4 to 14.3) |  | 714 | 53 | 7.4 (5.7 to 9.6) |  | 773 | 27 | 3.5 (2.4 to 5.0) | |
| p(trend) |  |  | 0.567 |  |  |  | 0.735 |  |  |  | 0.225 | |
| Prime definition: Responses to “I often feel very lonely’ including 5 (Somewhat agree) + 6 (Agree) + 7 (Strongly agree)  Sensitivity 1: Responses to “I often feel very lonely’ including 6 (Agree) + 7 (Strongly agree)  Sensitivity 2: Responses to “I often feel very lonely’ including 7 (Strongly agree) only | | | | | | | | | | | |  |

**Table S4: Pattern of cumulative relief from loneliness of contrasting definitions across green space strata**

|  |  |  |  |  |  |  |  |  |  |  |  |
| --- | --- | --- | --- | --- | --- | --- | --- | --- | --- | --- | --- |
| Green space | Cumulative relief from loneliness (prime definition) | | |  | Cumulative relief from loneliness (sensitivity 1) | | |  | Cumulative relief from loneliness (sensitivity 2) | | |
|  | N total | N cumulative relief | % (95% Confidence Interval) |  | N total | N cumulative relief | % (95% Confidence Interval) |  | N total | N cumulative relief | % (95% Confidence Interval) |
| Total | 1,282 | 733 | 57.2 (54.4 to 59.9) |  | 729 | 479 | 65.7 (62.2 to 69.1) |  | 284 | 206 | 72.5 (67.0 to 77.4) |
| Percent green space within 1600m | | | | | | | | | | | |
| 0-10% | 463 | 264 | 57.0 (52.5 to 61.5) |  | 246 | 166 | 67.5 (61.4 to 73.1) |  | 100 | 72 | 72.0 (62.3 to 80.0) |
| 10-20% | 496 | 285 | 57.5 (53.1 to 61.8) |  | 288 | 187 | 64.9 (59.2 to 70.2) |  | 109 | 80 | 73.4 (64.2 to 80.9) |
| 20-30% | 198 | 112 | 56.6 (49.6 to 63.3) |  | 120 | 77 | 64.2 (55.2 to 72.3) |  | 42 | 31 | 73.8 (58.3 to 85.0) |
| >30% | 125 | 72 | 57.6 (48.7 to 66.0) |  | 75 | 49 | 65.3 (53.8 to 75.3) |  | 33 | 23 | 69.7 (51.9 to 83.1) |
| p(trend) |  |  | 0.996 |  |  |  | 0.908 |  |  |  | 0.974 |
| Percent green space within 800m | | | | | | | | | | | |
| 0-10% | 541 | 310 | 57.3 (53.1 to 61.4) |  | 305 | 203 | 66.6 (61.1 to 71.6) |  | 120 | 90 | 75.0 (66.4 to 82.0) |
| 10-20% | 413 | 229 | 55.4 (50.6 to 60.2) |  | 246 | 155 | 63.0 (56.8 to 68.8) |  | 96 | 66 | 68.8 (58.7 to 77.3) |
| 20-30% | 179 | 109 | 60.9 (53.5 to 67.8) |  | 95 | 69 | 72.6 (62.8 to 80.7) |  | 39 | 31 | 79.5 (63.7 to 89.5) |
| >30% | 149 | 85 | 57.0 (49.0 to 64.8) |  | 83 | 52 | 62.7 (51.7 to 72.4) |  | 29 | 19 | 65.5 (46.5 to 80.6) |
| p(trend) |  |  | 0.678 |  |  |  | 0.353 |  |  |  | 0.437 |
| Percent green space within 400m | | | | | | | | | | | |
| 0-10% | 722 | 407 | 56.4 (52.7 to 60.0) |  | 426 | 278 | 65.3 (60.6 to 69.6) |  | 167 | 123 | 73.7 (66.4 to 79.8) |
| 10-20% | 273 | 160 | 58.6 (52.7 to 64.3) |  | 147 | 100 | 68.0 (60.0 to 75.1) |  | 57 | 40 | 70.2 (57.0 to 80.7) |
| 20-30% | 145 | 85 | 58.6 (50.4 to 66.4) |  | 74 | 48 | 64.9 (53.3 to 74.9) |  | 37 | 24 | 64.9 (48.1 to 78.6) |
| >30% | 142 | 81 | 57.0 (48.7 to 65.0) |  | 82 | 53 | 64.6 (53.7 to 74.3) |  | 23 | 19 | 82.6 (61.1 to 93.5) |
| p(trend) |  |  | 0.909 |  |  |  | 0.929 |  |  |  | 0.470 |
| Prime definition: Responses to “I often feel very lonely’ including 5 (Somewhat agree) + 6 (Agree) + 7 (Strongly agree)  Sensitivity 1: Responses to “I often feel very lonely’ including 6 (Agree) + 7 (Strongly agree)  Sensitivity 2: Responses to “I often feel very lonely’ including 7 (Strongly agree) only | | | | | | | | | | | |

**Table S5: Influence of green space on cumulative incidence of loneliness (sample 1)**

|  |  |  |  |  |  |  |
| --- | --- | --- | --- | --- | --- | --- |
|  | Model 1 | Model 2 | Model 3 | Model 4 | Model 5 | Model 6 |
|  | Odds Ratio (95% Confidence Interval) | | | | |  |
| Age group (ref: 15-24) | | | | | |  |
| 25-34 | 1.256 (0.964, 1.636) | 1.198 (0.910, 1.577) | 1.257 (0.965, 1.637) | 1.197 (0.910, 1.576) | 1.262 (0.969, 1.644) | 1.200 (0.912, 1.579) |
| 35-44 | 1.215 (0.923, 1.598) | 1.176 (0.886, 1.560) | 1.215 (0.923, 1.598) | 1.174 (0.885, 1.558) | 1.212 (0.921, 1.594) | 1.172 (0.883, 1.554) |
| 45-54 | 1.124 (0.858, 1.474) | 1.010 (0.764, 1.336) | 1.128 (0.860, 1.478) | 1.011 (0.765, 1.336) | 1.130 (0.862, 1.481) | 1.013 (0.766, 1.338) |
| 55-64 | 0.818 (0.603, 1.110) | 0.647 (0.470, 0.891) | 0.819 (0.604, 1.110) | 0.646 (0.469, 0.890) | 0.821 (0.605, 1.113) | 0.647 (0.470, 0.890) |
| 65-74 | 0.895 (0.642, 1.248) | 0.572 (0.397, 0.825) | 0.891 (0.639, 1.243) | 0.568 (0.394, 0.819) | 0.889 (0.638, 1.240) | 0.566 (0.393, 0.816) |
| 75+ | 1.400 (0.973, 2.016) | 0.800 (0.531, 1.204) | 1.397 (0.971, 2.011) | 0.795 (0.528, 1.197) | 1.394 (0.969, 2.005) | 0.793 (0.527, 1.193) |
| Sex (ref: Male) | | | | | |  |
| Female | 1.133 (0.973, 1.318) | 1.106 (0.948, 1.290) | 1.133 (0.974, 1.319) | 1.106 (0.948, 1.291) | 1.133 (0.974, 1.319) | 1.106 (0.948, 1.291) |
| Children (<15y) in the household (ref: No) | | | | | |  |
| Yes | 1.166 (0.971, 1.402) | 1.133 (0.941, 1.365) | 1.165 (0.969, 1.399) | 1.131 (0.939, 1.362) | 1.166 (0.970, 1.400) | 1.131 (0.939, 1.362) |
| Living circumstance - baseline only (ref: Married or cohabiting throughout) | | | | | |  |
| Married or cohabiting, then living alone | 2.879 (2.133, 3.887) | 2.665 (1.968, 3.609) | 2.865 (2.123, 3.868) | 2.653 (1.959, 3.592) | 2.884 (2.137, 3.892) | 2.663 (1.966, 3.606) |
| Living alone, then married or cohabiting | 0.843 (0.571, 1.244) | 0.700 (0.470, 1.041) | 0.840 (0.570, 1.240) | 0.697 (0.469, 1.037) | 0.844 (0.572, 1.244) | 0.699 (0.470, 1.040) |
| living alone throughout | 2.148 (1.784, 2.587) | 1.810 (1.488, 2.203) | 2.150 (1.785, 2.588) | 1.809 (1.487, 2.201) | 2.159 (1.793, 2.599) | 1.814 (1.491, 2.207) |
| Percentage green space (10% units) |  |  |  |  |  |  |
| within 1600m | 0.910 (0.847, 0.977) | 0.927 (0.862, 0.996) |  |  |  |  |
| within 800m |  |  | 0.944 (0.885, 1.006) | 0.957 (0.897, 1.021) |  |  |
| within 400m |  |  |  |  | 0.997 (0.944, 1.053) | 1.007 (0.953, 1.063) |
| Highest educational qualifications (ref: < Year 12) | | | | | |  |
| Year 12 to Adv. Dip. |  | 0.998 (0.821, 1.212) |  | 0.997 (0.821, 1.211) |  | 0.994 (0.819, 1.208) |
| University |  | 0.822 (0.650, 1.040) |  | 0.821 (0.649, 1.039) |  | 0.822 (0.650, 1.040) |
| Annual household income (ref: 0-$30k) | | | | | |  |
| $30k-$40k |  | 1.162 (0.830, 1.626) |  | 1.163 (0.831, 1.627) |  | 1.166 (0.833, 1.632) |
| $40k-$50k |  | 0.949 (0.663, 1.357) |  | 0.955 (0.668, 1.365) |  | 0.951 (0.665, 1.359) |
| $50k-$60k |  | 0.748 (0.520, 1.076) |  | 0.753 (0.524, 1.082) |  | 0.754 (0.524, 1.083) |
| $60k-$80k |  | 0.702 (0.499, 0.987) |  | 0.704 (0.501, 0.990) |  | 0.704 (0.501, 0.990) |
| $80k-$100k |  | 0.696 (0.494, 0.981) |  | 0.695 (0.493, 0.978) |  | 0.692 (0.492, 0.975) |
| $100k-$125k |  | 0.648 (0.458, 0.915) |  | 0.654 (0.463, 0.924) |  | 0.652 (0.462, 0.922) |
| 125k+ |  | 0.635 (0.468, 0.860) |  | 0.633 (0.467, 0.858) |  | 0.633 (0.467, 0.858) |
| % of last 12 months spent unemployed (ref: 0%) | | | | | |  |
| 1-24% |  | 0.818 (0.545, 1.226) |  | 0.816 (0.544, 1.224) |  | 0.816 (0.544, 1.224) |
| 25-49% |  | 0.932 (0.544, 1.598) |  | 0.928 (0.541, 1.589) |  | 0.931 (0.543, 1.595) |
| 50-74% |  | 0.608 (0.297, 1.244) |  | 0.598 (0.293, 1.224) |  | 0.596 (0.291, 1.219) |
| 75-100% |  | 1.042 (0.623, 1.742) |  | 1.042 (0.623, 1.742) |  | 1.041 (0.623, 1.740) |
| Disability or long-term health condition (ref: No) | | | | | |  |
| Yes |  | 1.509 (1.261, 1.805) |  | 1.511 (1.263, 1.807) |  | 1.512 (1.264, 1.809) |
| Area-level socioeconomic circumstances (deciles) |  | 1.033 (1.000, 1.067) |  | 1.034 (1.001, 1.067) |  | 1.036 (1.004, 1.070) |
|  |  |  |  |  |  |  |

**Table S6: Influence of green space on cumulative relief from loneliness (sample 2)**

|  |  |  |  |  |  |  |
| --- | --- | --- | --- | --- | --- | --- |
|  | Model 1 | Model 2 | Model 3 | Model 4 | Model 5 | Model 6 |
|  | Odds Ratio (95% Confidence Interval) | | | | |  |
| Age group (ref: 15-24) | | | | | |  |
| 25-34 | 0.730 (0.484, 1.102) | 0.816 (0.527, 1.262) | 0.731 (0.484, 1.102) | 0.816 (0.528, 1.263) | 0.730 (0.484, 1.101) | 0.815 (0.527, 1.261) |
| 35-44 | 0.641 (0.423, 0.970) | 0.723 (0.467, 1.120) | 0.644 (0.425, 0.975) | 0.726 (0.469, 1.125) | 0.643 (0.425, 0.974) | 0.725 (0.468, 1.123) |
| 45-54 | 0.649 (0.435, 0.968) | 0.747 (0.490, 1.138) | 0.650 (0.436, 0.969) | 0.748 (0.491, 1.139) | 0.650 (0.436, 0.969) | 0.747 (0.490, 1.138) |
| 55-64 | 0.611 (0.397, 0.939) | 0.762 (0.481, 1.205) | 0.610 (0.397, 0.937) | 0.762 (0.481, 1.205) | 0.609 (0.396, 0.936) | 0.760 (0.481, 1.203) |
| 65-74 | 0.898 (0.546, 1.477) | 1.297 (0.747, 2.250) | 0.901 (0.547, 1.483) | 1.302 (0.750, 2.259) | 0.896 (0.545, 1.475) | 1.296 (0.747, 2.248) |
| 75+ | 1.285 (0.679, 2.432) | 1.825 (0.913, 3.646) | 1.291 (0.682, 2.443) | 1.834 (0.918, 3.665) | 1.290 (0.682, 2.442) | 1.831 (0.916, 3.658) |
| Sex (ref: Male) | | | | | |  |
| Female | 0.941 (0.747, 1.185) | 0.933 (0.736, 1.182) | 0.938 (0.745, 1.181) | 0.931 (0.735, 1.180) | 0.939 (0.745, 1.182) | 0.931 (0.735, 1.180) |
| Children (<15y) in the household (ref: No) | | | | | |  |
| Yes | 0.920 (0.689, 1.229) | 0.891 (0.663, 1.197) | 0.919 (0.688, 1.228) | 0.890 (0.662, 1.196) | 0.917 (0.687, 1.226) | 0.889 (0.661, 1.195) |
| Living circumstance - baseline only (ref: Married or cohabiting throughout) | | | | | |  |
| Married or cohabiting, then living alone | 0.450 (0.281, 0.720) | 0.495 (0.305, 0.802) | 0.449 (0.280, 0.719) | 0.494 (0.305, 0.801) | 0.449 (0.280, 0.720) | 0.494 (0.305, 0.801) |
| Living alone, then married or cohabiting | 1.206 (0.706, 2.061) | 1.396 (0.798, 2.441) | 1.201 (0.703, 2.053) | 1.390 (0.795, 2.431) | 1.196 (0.701, 2.043) | 1.386 (0.793, 2.421) |
| living alone throughout | 0.473 (0.363, 0.617) | 0.523 (0.393, 0.696) | 0.474 (0.363, 0.618) | 0.524 (0.393, 0.697) | 0.473 (0.362, 0.617) | 0.523 (0.393, 0.696) |
| Percentage green space (10% units) |  |  |  |  |  |  |
| within 1600m | 1.036 (0.937, 1.147) | 1.027 (0.927, 1.139) |  |  |  |  |
| within 800m |  |  | 1.020 (0.929, 1.120) | 1.015 (0.922, 1.116) |  |  |
| within 400m |  |  |  |  | 1.013 (0.935, 1.097) | 1.008 (0.929, 1.094) |
| Highest educational qualifications (ref: < Year 12) | | | | | |  |
| Year 12 to Adv. Dip. |  | 1.169 (0.879, 1.554) |  | 1.169 (0.879, 1.554) |  | 1.169 (0.879, 1.555) |
| University |  | 1.125 (0.792, 1.599) |  | 1.123 (0.790, 1.596) |  | 1.125 (0.792, 1.600) |
| Annual household income (ref: 0-$30k) | | | | | |  |
| $30k-$40k |  | 0.963 (0.580, 1.599) |  | 0.961 (0.579, 1.596) |  | 0.962 (0.580, 1.598) |
| $40k-$50k |  | 0.920 (0.556, 1.520) |  | 0.924 (0.559, 1.526) |  | 0.924 (0.559, 1.527) |
| $50k-$60k |  | 1.011 (0.607, 1.683) |  | 1.012 (0.607, 1.684) |  | 1.012 (0.607, 1.685) |
| $60k-$80k |  | 0.864 (0.549, 1.362) |  | 0.865 (0.549, 1.363) |  | 0.865 (0.549, 1.363) |
| $80k-$100k |  | 0.730 (0.446, 1.195) |  | 0.730 (0.446, 1.194) |  | 0.730 (0.446, 1.194) |
| $100k-$125k |  | 1.371 (0.840, 2.239) |  | 1.370 (0.839, 2.237) |  | 1.368 (0.838, 2.233) |
| 125k+ |  | 1.303 (0.836, 2.030) |  | 1.303 (0.836, 2.031) |  | 1.303 (0.836, 2.031) |
| % of last 12 months spent unemployed (ref: 0%) | | | | | |  |
| 1-24% |  | 0.661 (0.383, 1.141) |  | 0.662 (0.384, 1.142) |  | 0.661 (0.383, 1.139) |
| 25-49% |  | 1.409 (0.756, 2.624) |  | 1.408 (0.756, 2.625) |  | 1.402 (0.752, 2.612) |
| 50-74% |  | 0.912 (0.381, 2.184) |  | 0.913 (0.381, 2.187) |  | 0.911 (0.380, 2.182) |
| 75-100% |  | 1.096 (0.625, 1.921) |  | 1.103 (0.630, 1.933) |  | 1.105 (0.630, 1.935) |
| Disability or long-term health condition (ref: No) | | | | | |  |
| Yes |  | 0.654 (0.506, 0.845) |  | 0.652 (0.505, 0.843) |  | 0.653 (0.505, 0.844) |
| Area-level socioeconomic circumstances (ref: decile) |  | 0.979 (0.937, 1.023) |  | 0.979 (0.936, 1.023) |  | 0.978 (0.936, 1.023) |
|  |  |  |  |  |  |  |

**Table S7: Effect modification of association between green space within 1600m and cumulative incidence of, and relief from, loneliness, by age group, cohabitation status, sex, and disability status**

|  |  |  |  |
| --- | --- | --- | --- |
|  | Cumulative incidence of loneliness |  | Cumulative relief from loneliness |
|  | Odds Ratio (95% Confidence Interval) | | |
| **Interaction 1: Green space x age group** |  |  |  |
| Age group (ref: 15-24) |  |  |  |
| 25-34 | 1.172 (0.760 to 1.807) |  | 0.803 (0.392 to 1.645) |
| 35-44 | 1.053 (0.684 to 1.619) |  | 0.754 (0.379 to 1.499) |
| 45-54 | 1.034 (0.667 to 1.603) |  | 0.593 (0.299 to 1.176) |
| 55-64 | 0.822 (0.500 to 1.351) |  | 1.058 (0.513 to 2.180) |
| 65-74 | 0.563 (0.326 to 0.973) |  | 0.830 (0.349 to 1.974) |
| 75+ | 1.051 (0.564 to 1.961) |  | 2.771 (0.904 to 8.488) |
| Percentage green space (10% units) | 0.937 (0.790 to 1.111) |  | 1.019 (0.770 to 1.347) |
| Green space x 25-34 | 1.016 (0.804 to 1.282) |  | 1.012 (0.685 to 1.494) |
| Green space x 35-44 | 1.075 (0.863 to 1.338) |  | 0.981 (0.690 to 1.393) |
| Green space x 45-54 | 0.984 (0.775 to 1.250) |  | 1.169 (0.812 to 1.682) |
| Green space x 55-64 | 0.837 (0.630 to 1.113) |  | 0.793 (0.538 to 1.169) |
| Green space x 65-74 | 1.009 (0.770 to 1.322) |  | 1.364 (0.847 to 2.197) |
| Green space x 75+ | 0.828 (0.591 to 1.162) |  | 0.770 (0.446 to 1.329) |
| p(trend) | 0.554 |  | 0.223 |
| **Interaction 2: Green space x cohabitation status** |  |  |  |
| Living circumstance - baseline only (ref: Married or cohabiting throughout) |  |  |  |
| Married or cohabiting, then living alone | 1.736 (1.045 to 2.884) |  | 0.541 (0.243 to 1.203) |
| Living alone, then married or cohabiting | 0.590 (0.318 to 1.092) |  | 1.062 (0.437 to 2.580) |
| living alone throughout | 2.110 (1.577 to 2.823) |  | 0.499 (0.323 to 0.771) |
| Percentage green space (10% units) | 0.933 (0.849 to 1.027) |  | 1.011 (0.872 to 1.172) |
| Green space x Married or cohabiting, then living alone | 1.316 (1.023 to 1.692) |  | 0.938 (0.602 to 1.461) |
| Green space x Living alone, then married or cohabiting | 1.119 (0.825 to 1.518) |  | 1.250 (0.700 to 2.230) |
| Green space x living alone throughout | 0.892 (0.763 to 1.043) |  | 1.030 (0.830 to 1.280) |
| p(trend) | 0.030 |  | 0.863 |
| **Interaction 3: Green space x gender** |  |  |  |
| Sex (ref: Male) |  |  |  |
| Female | 1.014 (0.789 to 1.305) |  | 0.907 (0.609 to 1.352) |
| Percentage green space (10% units) | 0.897 (0.806 to 0.997) |  | 1.016 (0.859 to 1.201) |
| Green space x Female | 1.062 (0.924 to 1.219) |  | 1.019 (0.824 to 1.260) |
| p(trend) | 0.397 |  | 0.864 |
| **Interaction 4: Green space x disability or long-term health condition** |  |  |  |
| Disability or long-term health condition (ref: No) |  |  |  |
| Yes | 1.287 (0.972 to 1.704) |  | 0.610 (0.407 to 0.913) |
| Percentage green space (10% units) | 0.897 (0.823 to 0.977) |  | 1.010 (0.889 to 1.148) |
| Green space x Yes | 1.116 (0.963 to 1.295) |  | 1.050 (0.845 to 1.303) |
| p(trend) | 0.145 |  | 0.661 |
|  |  |  |  |
